# Emergency blood transfusion practices among anaemic children presenting to an urban emergency department of a tertiary hospital in Tanzania

**DOI:** 10.1186/s12878-017-0091-y

**Published:** 2017-11-10

**Authors:** Catherine R. Shari, Hendry R. Sawe, Brittany L. Murray, Victor G. Mwafongo, Juma A. Mfinanga, Michael S. Runyon

**Affiliations:** 1 Emergency Medicine Department Muhimbili University of Health and Allied Sciences, P.O Box 65001, Dar es Salaam, Tanzania; 2 Emergency Medicine Department, Muhimbili National Hospital, Dar Es Salaam, Tanzania; 3 Division of Pediatric Emergency Medicine, Emory University School of Medicine, Emory University, Atlanta, GA USA; 4 Department of Emergency Medicine, Carolinas Medical Center, Charlotte, NC USA

**Keywords:** Anaemia, Emergency blood transfusion, Emergency medicine department, Paediatric, Tanzania

## Abstract

**Background:**

Severe anaemia contributes significantly to mortality, especially in children under 5 years of age. Timely blood transfusion is known to improve outcomes. We investigated the magnitude of anaemia and emergency blood transfusion practices amongst children under 5 years presenting to the Emergency Department (ED) of Muhimbili National Hospital (MNH) in Tanzania.

**Methods:**

This prospective observational study enrolled children under 5 years old with anaemia, over a 7-week period in August and September of 2015. Anaemia was defined as haemoglobin of <11 g/dL. Demographics, anaemia severity, indications for transfusion, receipt of blood, and door to transfusion time were abstracted from the charts using a standardized data entry form. Anaemia was categorized as severe (Hb <7 g/dL), moderate (Hb 7–9.9 g/dL) or mild (Hb 10–10.9 g/dL).

**Results:**

We screened 777 children, of whom 426 (55%) had haemoglobin testing. Test results were available for 388/426 (91%), 266 (69%) of whom had anaemia. Complete data were available for 257 anaemic children, including 42% (*n* = 108) with severe anaemia, 40% (*n* = 102) with moderate anaemia and 18% (*n* = 47) with mild anaemia. Forty-nine percent of children with anaemia (*n* = 125) had indications for blood transfusion, but only 23% (29/125) were transfused in the ED. Among the non-transfused, the provider did not identify anaemia in 42% (*n* = 40), blood was not ordered in 28% (*n* = 27), and blood was ordered, but not available in 30% (*n* = 29). The median time to transfusion was 7.8 (interquartile range: 1.9) hours. Mortality was higher for the children with severe anemia who were not transfused as compared with those with severe anaemia who were transfused (29% vs 10%, *p* = 0.03).

**Conclusion:**

The burden of anaemia is high among children under 5 presenting to EMD-MNH. Less than a quarter of children with indications for transfusion receive it in the EMD, the median time to transfusion is nearly 8 h, and those not transfused have nearly a 3-fold higher mortality. Future quality improvement and research efforts should focus on eliminating barriers to timely blood transfusion.

**Electronic supplementary material:**

The online version of this article (10.1186/s12878-017-0091-y) contains supplementary material, which is available to authorized users.

## Background

Anaemia is a significant contributor to mortality and morbidity globally, especially in children under 5 years old [1, 2]. It disproportionately affects children in Sub Saharan Africa [2]. In low income countries such as South East Asia and West Africa, anaemia has remained to be a significant health problem with even higher rates of mortality [3, 4]. In East Africa, anaemia is estimated to affect more than three quarters of children under 5 years [5, 6], and studies in Tanzania have shown even higher rates of anaemia [7–9].

Management of anaemia varies depending on the underlying etiology and severity, but in cases of severe and life threatening anaemia, blood transfusion has remained the most critical lifesaving intervention and is shown to improve outcomes [10, 11]. Studies have shown that blood transfusion is most beneficial when given early and that delayed transfusion leads to increased mortality [10, 12]. It has also been shown that with delay in treatment of severe anaemia, irreversible tissue damage can occur and patients may suffer morbidity that can persist for months after their initial treatment [13]. Therefore, early recognition and treatment of children with severe anaemia is vital to optimizing outcomes.

The WHO has produced guidelines for blood transfusion in severe anaemia; however, many children requiring transfusion under these guidelines do not receive blood. The availability of blood in emergencies is still a challenge in many developing countries [14]. The blood supply is not adequate to meet population demands in most of the low income countries such as Bangladesh, South East Asia, South and West Africa [3, 4, 15–18]. This lack of blood has been attributed to limited emergency and critical care services in these areas [19, 20]. In 2014, the National Blood and Transfusion Services (NBTS) in Tanzania estimated that the need for blood is about 450,000 units yearly, but only a third of that amount is collected [14]. Hence, the blood supply does not meet the population demand for blood transfusion [21]. In low income countries, adherence to WHO guidelines to transfusion is poor and most patients requiring blood do not get it in appropriate manner [12]. At Muhimbili National Hospital (MNH) in Dar es Salaam, Tanzania, emergency blood transfusion in the Emergency Department (ED) for those meeting the WHO guidelines is the expected standard of clinical practice. However, some patients who present with anaemia and indications for blood transfusion do not receive it in a timely manner. As a result, some patients die without being transfused. The emergency blood transfusion practices among anaemic children less than 5 years old presenting to the MNH-ED has not been previously studied. We aimed to assess the burden of anaemia in the children arriving at the MNH-ED, evaluate the emergency blood transfusion practices at our hospital, and report the outcomes of children with anaemia.

## Methods

This was a prospective observational study of anaemic children who presented to the MNH-ED in August and September of 2015. MNH is located in Dar es Salaam, Tanzania and is the largest tertiary referral hospital and the main medical teaching hospital in the country. The MNH-ED was opened in 2010 and is the first and only 24-h/day full capacity ED in the country, attending to an average of 150 to 200 patients daily. Approximately 13% of the patients are children under the age of 5 years. The ED is staffed with seven medical doctors (registrars and residents) and 20 nurses who work under supervision of two emergency specialist and critical care nurses.

All children age 1 month to 5 years were consecutively screened for inclusion in the study. Laboratory testing was according to standard clinical care at the discretion of the treating doctor as part of standard care. Children found to be anaemic (laboratory confirmation of Hb < 11 g/dl) [22], were enrolled in the study. Signed, informed consent was obtained from the children’s parent(s) or guardians. The study excluded children whose parent(s) or guardian(s) did not consent, children who were declared dead on arrival to the ED and those with incomplete or unavailable charts.

Patient screening and enrollment was performed by the principal investigator and one trained research assistant. Study data were recorded on a structured case report form. The indications for blood transfusion were as defined by WHO [22] and include:

1. Haemoglobin level less than 4 g/dl, or
2. Haemoglobin level of 4-7 g/dl with any of the following: shock, clinically detectable dehydration, impaired consciousness, respiratory acidosis revealed by deep labored breathing, heart failure, or more than 20% of red blood cells parasitized by malaria parasite.
3. Haemoglobin levels more than 4 g/dl with continuing bleeding

Our primary outcome was the proportion of anaemic children with WHO indications who received transfusion in the ED. Secondary outcomes included demographics, prevalence and severity of anaemia, variability in the indications for blood transfusion, time to blood transfusion, reasons for delays in transfusion, and in-hospital mortality. Age adjusted tachycardia was defined as heart rate > 180 beats/min in children less than 2 years and >140 beats/min in children aged 2–5 years, while age adjusted tachypnea was defined as respiratory rate > 34 breaths/min in children less than 2 years and >22 breaths/min in children 2–5 years.

Data are summarized with descriptive statistics, including the counts and percentages and medians and interquartile ranges (IQR). Categorical variables are presented as frequencies and percentages, and continuous variables are presented as medians and interquartile ranges (IQR). Ninety-five percent confidence intervals (CI) are presented where appropriate. The chi square test or Fisher’s exact test were used to compare categorical variables and the Mann-Whitney U-test was used to compare continuous variables. Data were analysed using Microsoft Excel 2013 (Microsoft corporation, Redmond, WA, USA), Stata (version 13, StataCorp LP, Texas, USA), and StatsDirect (version 3.0.167, StatsDirect Ltd., Cheshire, UK).

Ethical clearance was obtained from the Research and Publications Committee of MUHAS and director of medical services of MNH.

## Results

**Fig. 1:**
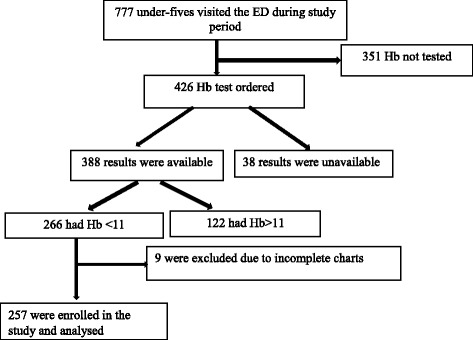
Patient screening process (flow diagram). This flow diagram shows study screening and enrollment

We screened 777 eligible children, representing 100% of children seen at the EMD during the study period. Of these, 426 (55%) had a haemoglobin level ordered, 388/426 (91%) had available results, and more than two thirds of all patients tested (68.6%, 95% CI: 63.7–73.1%) were found to be anaemic (Hb <11 g/dl) (Fig. 1).

### Demographics and classification of anaemia

Of the 266 patients with anaemia, 257 (97%) had complete data for the primary outcome and were included in the analysis. The median age was 16 (IQR 8–31) months, 158 (61.5%) were male, and 108 (42%) had severe anaemia. There was no difference in the age or gender distribution among those children with and without transfusion indications. As expected, more children with severe anaemia had transfusion indications. Most patients (196; 76.3%) were referred from hospitals across Dar es Salaam and mainland Tanzania and those patients were more likely to have transfusion indications. (Table 1). The most common diagnoses were malaria (20.1%) and sickle cell disease (18.3%). Other diagnoses included sepsis (7.6%), pneumonia (7.3%), and malnutrition (5.4%). The most common chief complaints were fever (26%), vomiting (8.3%), cough (8.1%), general body malaise (7.6%), difficulty in breathing (6.5%), and diarrhea (5.7%).

**Table 1:** Demography and severity of anaemia^a^

Demographic characteristics	All (*N* = 257)	Indications (*N* = 125)	No indications (*N* = 132)	*P*-value
Gender				0.6
Male	158 (61.5%)	75 (60%)	83 (62.9%)	
Female	99 (38.5%)	50 (40%)	49 (37.1%)	
Age (months)	16 (8–31)	14 (6.5–26)	18 (9–34)	0.07
Referral				<0.0001
Patient presenting from home	61 (23.7%)	13 (10.4%)	48 (36.4%)	
Referral from another healthcare facility	196 (76.3%)	112 (89.6%)	84 (63.6%)	
Severity of anaemia				<0.0001
Mild	47 (18.3%)	0	47 (35.6%)	
Moderate	102 (39.7%)	24(19.2%)	78 (59.1%)	
Severe	108 (42.0%)	101 (80.8%)	7 (5.3%)	
Vitals				
Heart rate (beats/min)	147 (131–164)	151 (136–166)	143.5 (124–161.5)	0.02
Age-adjusted tachycardia^d^	58 (22.5%)	40 (32%)	29 (22%)	0.02
Respiratory rate (breath/min)	33 (29–38)	34 (30–49)	33 (28–36)	0.08
Age-adjusted tachypnea^b^	160 (64.3%)	59 (47.2%)	82 (62.1%)	0.01
SPO2% < 95%	23 (8.9%)	12 (9.6%)	11 (8.3%)	0.89
Temperature % < 36, > 38^c^	49 (20.2%)	29 (21.6%)	20 (15.2%)	0.21

^a^Data are summarized as counts (percentage) or median (interquartile range)

^b^Respiratory rate data were missing for 8 patients

^c^Temperature data were missing for 38 patients

^d^Age adjusted tachycardia was defined as heart rates >180 beats/min in children less than 2 years and >140 beats/min in children aged 2–5 years, while the age adjusted tachypnea was defined as respiratory rates >34 breaths/min in children less than 2 years and >22 breaths/min in children 2–5 years. (Cited from International paediatric sepsis consensus conference: definition for sepsis and organ dysfunction in paediatrics)

Table 1 shows overall patient demographics, severity of anaemia and comparison of these characteristics between those with indications for transfusion and those with no indications for transfusion

### Indications for blood transfusion and transfusion status

Overall, 125 (48.6%) children had WHO-defined indications for blood transfusion at the time of ED presentation. Of these children, 45 (36%) had Hb <4 g/dl, 76 (60.8%) had Hb between 4 g/dl and 7 g/dl with shock, and 4 (3.2%) had Hb > 4 g/dl with continuous bleeding.

**Fig. 2:**
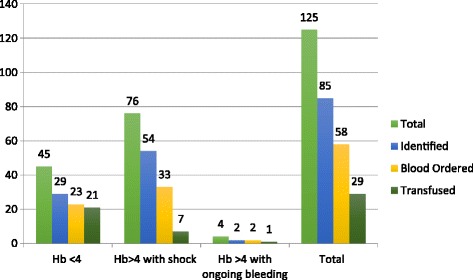
Indications and transfusion status bar chart. Indicates number of children under 5 years old with indications for transfusion on the vertical axis and the horizontal axis indicates the transfusion indication categories as per WHO guidelines

Emergency physicians identified anaemia in 85 (68%) of the children with WHO-defined indications for blood transfusion, and ordered blood in 58 (68%). Blood was transfused in 29 (23.2%) of those with indications (Fig. 2).

### Timing of blood processing and transfusion

The median ‘door to transfusion’ time (the time from arrival in the ED resuscitation room to the time transfusion started) was 468 min (IQR 410–525). The longest fraction of this time (190 min (IQR 180–280) was the point from when the blood tubes for cross matching were sent to the laboratory until the patients received the transfusion, while the shortest fraction of time (30 min IQR 25–60) was from the doctor’s order for crossmatch to the time that the samples was sent to the laboratory.

### Anaemia recognition and plan for blood transfusion

Among the 125 patients with WHO-defined indications for blood transfusion, 96 (76.8%) did not receive transfusion while in the ED. In 40 (41.7%) of these patients, the physicians did not document the clinical features of anaemia, its severity, or blood transfusion indications. In 27 (28.1%) of these patients anaemia was identified by the physician, but transfusion was not ordered, and in 29 (30.2%) of these patients, blood was ordered but it was unavailable.

### ED outcome and patients disposition

Among the 257 children with anaemia, most (197; 76.7%) were admitted to the general pediatrics wards. Only 1.6% (4/257) were sent to the advanced paediatric care unit (APCU), 15.6% (40/257) were admitted to the pediatric surgery ward, 3.9% (10/257) were discharged to home from the ED, and 2.3% (6/257) died in the ED.

### Overall patient outcomes and transfusion status

Among 241 Patients who were admitted to in-patient wards, 193 (80%) survived to discharge and 48 (20%) died while in the hospital.

Among 125 patients who had indications for blood transfusion at presentation to the ED, the overall mortality rates of those transfused and those not transfused at the ED were 10.3% and 29.2%, respectively (relative risk of 2.82, 95% CI 1.04–8.45). Table 2.

**Table 2:** Outcomes of anaemic under-fives with indications for BT (*N* = 125)

Outcome	Total *n* = 125 n (%) [95% CI]	Transfused in the ED *n* = 29 n (%) [95%CI]	Not Transfused in the ED *n* = 96 n (%) [95% CI]	*P*- value
ED mortality	6 (4.8%) (95%CI 1.05–8.55)	2 (6.9%) (95%CI −2.3-16.1)	4 (4.2%) (95% CI 0.19–8.21)	0.067
24 h mortality	19 (15.2%) (95% CI 8.91–21.49)	2 (6.9%) (95% CI −2.3-16.1)	17 (17.7%) (95% CI 10.1–25.3)	0.058
Overall mortality	31 (24.8%) (95% CI 17.23–32.37)	3 (10.3%) (95% CI −0.76-21.36)	28 (29.2%) (95% CI 20.01–38.19)	0.032

Table 2 shows the ED mortality, 24-h mortality, and overall mortality between the children with WHO indications for transfusion that received blood in the ED, and those that did not with their respective 95% confidence intervals

### Inpatient transfusion practices

Among the 96 children with indications for blood transfusion who were not transfused in the ED, 59.4% (*n* = 57) did receive a blood transfusion in the wards before the time of their discharge or death.

## Discussion

Our study has revealed a large burden of anaemia among children under the age of 5 years presenting to the MNH-ED. Since laboratory testing was at the discretion of the treating doctor, not all children were screened for anaemia. If we assume the unlikely scenario that anaemia was absent in all of the children who were not screened, we can conservatively estimate that at least one third (257/777) of children presenting to our ED are anaemic. We found that more than two thirds of all children tested in our ED have anaemia as compared to the estimated prevalence of anaemia globally (24.8%) [2], and that of developed countries [15, 16, 23, 24]. However, these findings were very similar to other low resource countries in South and Southeast Asia, and South and West Africa [3, 4, 16, 18]. The prevalence of anaemia in this study was lower compared to the study done in Ghana where more than a quarter of children admitted had anaemia, with as 71% of these children having severe anaemia which is higher compared to our study where children with severe anaemia comprised 42% of children with anaemia [4]. Our findings are also similar to the 2016 Tanzania Demographic and Health Survey (TDHS), which reported the prevalence of anaemia among children under 5 years-old to be 58% [25].

The proportion of anaemia found in our study is slightly lower than that previously reported among the MNH inpatient paediatric population, as documented by *Magesa* et al. who found a prevalence of 80.7% [8, 9]. Likewise, anaemia was found in 76% of children under 5 presenting to one of several hospitals in East Africa with severe infection [26]. The slightly lower rate of anemia in our cohort is likely due to the fact that we enrolled all children who underwent haemoglobin testing, regardless of their clinical condition or diagnosis. In fact, anemia was much more common in our cohort of children presenting to the ED than the 12% rate of anaemia reported in a study of 53,174 children admitted to one of 10 hospitals in Kenya [12].

The severity of anaemia in our study was striking. Although the proportion of anaemia in our study was similar to what is reported by TDHS [25], the distribution of severity of anaemia was very different. The proportion of anaemic under-fives with moderate to severe anaemia was higher (81.7%, 95% CI: 76.4–86.2%) in our population than the 32% rate of moderate or severe anaemia documented in the TDHS report [25]. This difference may reflect a different burden of disease that presents to MNH-ED. The patient population at MNH is largely referred from other hospitals, including 76.3% of our cohort, and these patients may be more likely to be anaemic and have transfusion indications than patients presenting to regional hospitals, either due to illness severity or simply because they were transferred to MNH specifically for blood transfusion due to lack of blood at the peripheral hospitals [2, 11]. The proportion of severe anaemia also raises the percentage of children meeting WHO transfusion criteria. Furthermore, the proportion of severe anaemia in our study is higher compared to other East African reports (12% in Kenya and 41% in Eastern Uganda) [26]. Despite the proportion of children with severe anaemia being high in our study (42%), it was lower compared to studies in Ghana where up to 71% present with severe anaemia [27]. The causes of anaemia in our study were found to be similar with a report from several Kenyan Hospitals where malaria was the leading cause of anaemia [12].

Our study showed that 48.6% of the anaemic under-fives presented at the ED with clear WHO defined indications for blood transfusion. Despite the high prevalence of anaemic under-fives with indications for transfusion in the MNH-ED, the transfusion rate was low (23.2%). This is quite low when compared to rates from EDs in the developed world [28]. We were unable to find data on transfusion rates in other ED settings in East Africa, but generally among paediatric inpatient populations in East Africa, transfusion rates have also been found to be low (20–45%), demonstrating poor adherence to WHO guidelines for transfusion [10, 26].

In our study, the low blood transfusion rate was associated with a number of different variables. The majority of patients who met transfusion criteria were not transfused because the physician either did not clearly diagnose anaemia or did not identify the indication for transfusion. This finding was in contrast to what was known in the past about physician gestalt. Although physicians in our ED have the ability to accurately diagnose anaemia based on clinical exam as reported by *Sawe* et al. [29], anaemia was not documented in many of the children in our study. There is a clear need for physicians to take time to properly examine children and look for signs of anaemia even when the primary chief complaint or referral diagnosis is not obviously related to anaemia or the need for blood transfusion. For example, in our setting some patients with complaints such as burn injuries and foreign bodies had severe anaemia with clear indications for blood transfusion. Moreover, when laboratory tests such as hemoglobin are often ordered, documentation of the results and their impact on clinical decision-making should be recorded in the physician documentation.

Physicians did not order blood transfusion in the ED for the majority of patients (67/125, 53.6%) who had indications according to the WHO guidelines. Even when transfusions were ordered in the ED, they were administered in only half of cases (29/58, 50%). This finding was similar to a study done in Kenya, where the majority of patients did not have an order for blood transfusion and 18% of those who did have an order did not receive the blood [12]. The reasons for these findings deserve further study. Possible reasons include both individual provider and system factors. For example, there may be a lack of clinical knowledge amongst providers regarding the criteria for ordering blood. Or, it could be due to the fact that blood and blood products continue to be a scarce resource at MNH and within Tanzania at large, as shown in previous NBTS reports [14]. According to the NBTS, only one third of the blood products that are needed in Tanzania each year are actually collected [14]. The scarcity of blood in Tanzania is not a novel finding. Blood has remained to be scarce in most developing countries and it significantly affect blood transfusion practices in most of these countries [4, 16, 18, 30]. Finally, some ED providers may find it inconvenient to order blood in the MNH-ED as it can increase the length of stay and contribute to overcrowding in the ED. This concern is illustrated in our study as the median time from door to transfusion is 7.8 (IQR 1.9) hours, which creates a backlog of patients in the resuscitation bay. It may have been hard for some physicians to accommodate these patients who needed to wait for blood for a very long time, especially during busy shifts and night shifts. Among those with indications for transfusion who did not receive it in the ED, only 60% were transfused after admission, further underscoring the importance of identifying and treating these children in the ED.

The median time interval to blood transfusion was very long in this study, nearly 8 h, with the blood processing at the laboratory taking the longest time interval. This may be explained by multiple possible factors including patients spending time on a queue to see a doctor, clearing registration/system issues, or in the laboratory for processing and preparation. This time interval is much longer than the guidelines from more mature healthcare systems, suggesting that uncross matched blood should ideally be available within 10 min, and group specific blood should be available within 30 min [31]. The specific cause of this delay in our department was not captured by our study methodology, but may include variables such as overcrowding in the ED, insufficient staff, a lack of blood products in the blood bank, or a lack of a systematic process to ensure availability of timely transfusion in the ED.

In this study, the overall mortality among patients with anaemia was 31/257 (12.1%, 95% CI: 8.6–16.6%), which is similar to prior reports from other locations in sub-Saharan Africa [32, 33]. The mortality rate among children who had indications for transfusion but were not transfused in the ED was almost three times higher than that of those who were transfused while in the ED (29.2% vs 10.3%, *p* = 0.032). Therefore, our study furthers the concept that early transfusion is associated with decreased mortality as found in other settings [13]. Our findings were similar to the study done by *Lackritz* et al. in Kenya among inpatient paediatric population, which showed a mortality benefit when blood was given early on the day of admission for those who presented with indications for blood transfusion as compared to those who received blood later [10]. However, the findings in our study did not take into account the other potential confounding factors that might contribute to the mortality difference.

The results from this study were disseminated internally to the practitioners at the MNH-ED and reasons for failure to identify anaemia and order indicated blood transfusions were explored. These included ED overcrowding necessitating a high patient turnover that did not allow time for physicians to wait for haemoglobin level results, impairing their ability to diagnose anaemia and make appropriate treatment decisions in a timely fashion. As a result of these findings, point of care haemoglobin testing was introduced to help physicians more rapidly diagnosis anaemia and initiate treatment. Furthermore, we provided targeted physician education on the clinical and laboratory assessment of anaemia, the WHO transfusion guidelines, and the importance of timely treatment in optimizing patients’ outcomes.

### Limitations

Our study limitations include that it was based at a single centre, which serves as the only full capacity ED in the country. This may limit the generalizability of our results. Another limitation is that we relied on physician documentation of anaemia and transfusion indications and this methodology may have underestimated recognition of these clinical diagnoses due to poor documentation. Despite this, our results are striking in that less than half of the patients with indications for transfusion had a transfusion order placed in the ED and only half of those for whom an order was placed were actually transfused. Finally, some paediatric patients presented to the ED during the study time period had no haemoglobin level measurements, which lead to their exclusion from the study; however, it is likely that some of those children were anaemic, resulting in an underestimation of the overall anaemia burden in our study population.

## Conclusion

The burden of anaemia is high among children under 5 years-old presenting to EMD-MNH. Less than a quarter of children with indications for transfusion receive it in the EMD, mostly due to issues around low rates of anaemia diagnosis, transfusion orders, and blood availability. The median time to transfusion is nearly 8 h and among those who are not transfused in the ED have a nearly 3-fold higher mortality. Future quality improvement and research efforts should focus on larger scale studies in multiple centres aimed at eliminating barriers to timely blood transfusion.

## Additional file

Additional file 1: A set of all data generated and analysed during this study. (XLSX 68 kb)

## Abbreviations

APCU: Acute Paediatric Care Unit
ED: Emergency Department
FBP: Full blood picture
Hb: Haemoglobin
MNH: Muhimbili National Hospital
MRN: Medical registration number
MUHAS: Muhimbili University of Health and Allied Sciences
NBTS: National Blood Transfusion Services
RBC: Red Blood Cell
TDHS: Tanzania Demographic and Health Surveys
US: United States of America
WHO: World Health Organization
